# Supplementary material for: Content-rich biological network constructed by mining PubMed abstracts
Source: BMC Bioinformatics. 2004 Oct 8;5:147. doi: 10.1186/1471-2105-5-147 (PMC528731; doi:10.1186/1471-2105-5-147)
Supplement: Additional File 2 — The original results of the above study (non-essential files are deleted to keep the file size under the limit set by BMC bioinformatics). [file 1471-2105-5-147-S2.bz2 › chilibotAdditionalFile2/dip10/102ID7937747E432/html/MCL1_BCL2.html]

 


 **MCL1** and **BCL2** 
  
Found 328 abstracts in PubMed, retrieved 10.  
 

 What does Google say? 
 PDF only 
| .edu only 

---

**Interactive relationship** (e.g. stimulation, inhibition, etc)

**Neutral relationship**- gossypol induced apoptosis triggers down regulation of anti apoptosis Bcl 2  [ **BCL2** ]  members Bcl X L, Bag 1 and Mcl 1  [ **MCL1** ] , up regulation of pro apoptosis Bcl 2  [ **BCL2** ]  member Bak, activation of caspase,, and 9, up regulation of Apaf 1, release of cytochrome c cyto c from mitochondria, and activation of both DFF45 and PARP.  Ref: 12818369 Biochem Pharmacol, 2003
- Here we show that Bid, a crucial proapoptotic protein that integrates the action of other Bcl 2  [ **BCL2** ]  family members, exhibits discrete specificity for metabolites of cardiolipin, especially monolysocardiolipin MCL  [ **MCL1** ] .  Ref: 12894218 Cell Death DifferCell Death Differ, 2003
- MCL  [ **MCL1** ]  may thus play a crucial role in connecting lipid metabolism, relocation of Bid to mitochondria and integrated action of Bcl 2  [ **BCL2** ]  proteins on mitochondrial membranes.  Ref: 12894218 Cell Death DifferCell Death Differ, 2003

**Non-interactive relationship** (e.g. studied together, co-existance, homology, etc.)

- In contrast, treatment of cells with LY294002, to inhibit phosphoinositide 3 kinase PI3K, caused downregulation of Bcl 2  [ **BCL2** ]  and Mcl 1  [ **MCL1** ]  and allowed DeltaMEKK1 ER\* to elicit a robust apoptotic response characterized by activation of Bax and caspases.  Ref: 12879014 Oncogene, 2003
- Expression of the anti apoptosis proteins, Bcl 2  [ **BCL2** ]  and Mcl 1  [ **MCL1** ]  expression decreased time dependently while Bax expression increased.  Ref: 12883267 Pancreas, 2003
- However, the expression levels of Bcl 2  [ **BCL2** ] , Bax, Bak and Mcl 1  [ **MCL1** ]  were unchanged.  Ref: 12842996 Blood< MedlineTA>Blood, 2003
- The stress induced transcription factor GADD153 and the  **Bcl2**  related  [ **MCL1** ]  protein Bak are upregulated in response to fenretinide.  Ref: 12880976 Cancer Lett, 2003
- Remarkably, these events occurred in the absence of any reduction in the expression of the Bcl 2  [ **BCL2** ]  family members Bcl 2  [ **BCL2** ] , Mcl 1  [ **MCL1** ]  and Bcl XL, or any change in the proapoptotic molecules Bad or Bax.  Ref: 12842827 Am J Physiol Gastrointest Liver Physiol< MedlineTA>Am J Physiol Gastrointest Liver Physiol, 2003
